# Changes in Anthropometric and Biochemical Parameters Following High-Protein and High-Density Enteral Nutrition in Chronically Ventilated Patients: A Retrospective Study

**DOI:** 10.3390/nu18132076

**Published:** 2026-06-24

**Authors:** Jenny Nahman Sichin, Lena Valetzky, Yosef Mishal, Oren Froy

**Affiliations:** 1 Bet Hadar Ashdod—Nursing & Rehabilitation Medical Center, Ashdod 7724221, Israel; 2 Barzilai Medical Center, Ashqelon 7830604, Israel; 3 Institute of Biochemistry, Food Science and Nutrition, Robert H. Smith Faculty of Agriculture, Food and Environment, The Hebrew University of Jerusalem, Rehovot 7610001, Israel

**Keywords:** albumin, body weight, nutritional support, clinical nutrition, enteral feeding

## Abstract

**Background/Objectives**: Nutritional support is a key component in the management of chronically ventilated patients, who are at high risk of malnutrition due to prolonged illness and metabolic stress. Enteral nutrition, particularly high-protein formulas (HPFs) and high-density formulas (HDFs), is commonly used to improve clinical outcomes; however, their effects on anthropometric and biochemical parameters remain incompletely understood. Our objective was to evaluate the association of HPFs and HDFs with changes in anthropometric and biochemical parameters in chronically ventilated patients receiving enteral nutrition. **Methods**: This retrospective study evaluated chronically ventilated patients receiving long-term enteral nutrition. Patients were categorized into four groups based on feeding strategy: continuous HPF, transition to HPF, transition from HPF and transition to HDF. Body weight, serum albumin and total protein were assessed at baseline and follow-up (up to 6 months). Within-group changes were analyzed using paired statistical tests. **Results**: Within-group analyses demonstrated changes in body weight, body mass index and serum albumin levels over time. Body weight increased significantly across all groups. The greatest increase was observed in patients transitioning to an HPF (70.82–75.35 kg, *p* = 0.00019), with a significant increase also following HDF administration (59.51–62.57 kg, *p* = 0.0389). Serum albumin increased significantly only in the transition-to-HPF group. HDF administration showed a non-significant increase in albumin and a near-significant rise in total protein. **Conclusions**: Enteral nutrition strategies were associated with changes in anthropometric and biochemical parameters in chronically ventilated patients. HPFs and HDFs were associated with improved body weight, with biochemical improvements most evident after HPF initiation and favorable trends observed with HDF administration. Future prospective studies with standardized protocols and objective nutritional markers are warranted.

## 1. Introduction

Malnutrition is highly prevalent among chronically ventilated patients and is associated with impaired immune function, delayed recovery, increased healthcare utilization and reduced quality of life [1]. Long-term mechanical ventilation is often accompanied by catabolic stress, muscle wasting and difficulty maintaining adequate nutritional intake [1]. Malnutrition remains a significant clinical challenge among patients requiring enteral nutrition, particularly in those with chronic illness, critical care needs, or impaired oral intake. Inadequate nutritional status is associated with increased morbidity, prolonged hospitalization, impaired immune function and higher mortality rates [2,3]. Early and appropriate nutritional support is therefore considered a cornerstone of critical care management.

Enteral nutrition represents the preferred method of nutritional support in these patients, as it preserves gastrointestinal function, supports gut integrity and reduces complications compared to parenteral nutrition [4]. Current guidelines advocate for early initiation of EN, ideally within 24–48 h of ICU admission, provided that there are no contraindications [5]. In chronically ventilated populations, enteral feeding is typically administered over prolonged periods, making optimization of formula composition particularly important [1]. Despite these recommendations, achieving optimal nutritional targets remains challenging in clinical practice due to feeding intolerance, interruptions and variability in prescription practices.

Standard enteral formulas aim to provide balanced macronutrient and micronutrient intake. However, their effectiveness may vary depending on patient-specific factors, such as metabolic demands, inflammatory status and underlying disease [6]. In critically ill and chronically ventilated patients, specialized formulas, including high-protein formulas (HPFs) and high-density formulas (HDFs), are frequently used to address these limitations. Protein provision has emerged as a key component of nutritional therapy, as adequate intake is essential to mitigate catabolism, preserve lean body mass, and support immune function [7]. Nevertheless, the optimal protein dose remains controversial, with some studies suggesting improved outcomes with higher protein intake, while others report inconsistent findings [8]. In response to these limitations, specialized enteral formulas have been developed to better address specific nutritional needs [4]. HPFs are designed to enhance protein delivery and support the preservation or restoration of lean body mass, particularly in patients with increased protein requirements or protein-energy malnutrition [9]. In contrast, HDFs provide a higher caloric content per unit volume, which may be beneficial in patients with fluid restrictions, reduced feeding tolerance, or increased energy demands [10]. Despite their growing clinical use, the relative contributions of protein enrichment versus caloric density to nutritional outcomes remain incompletely understood.

Assessment of nutritional status in chronically ventilated patients remains challenging. Common biochemical markers, such as albumin and total protein, are influenced by inflammation, hydration status and chronic disease burden, limiting their reliability as nutritional indicators [11]. Additional markers, including inflammatory parameters, such as C-reactive protein (CRP) or urinary nitrogen excretion, may provide complementary information but are not always consistently available in retrospective datasets [12]. Therefore, combining biochemical markers with anthropometric measurements, such as body weight, provides a more comprehensive evaluation of nutritional outcomes. Specialized enteral nutrition formulations may play a particularly important role in patients with increased nutritional requirements [13]. However, data regarding their effectiveness across different modes of administration, such as continuous use, initiation, or discontinuation, remain limited.

Despite growing research in this area, there remains a lack of real-world evidence evaluating how different enteral nutrition strategies influence both anthropometric and biochemical outcomes in clinical populations. In particular, limited data exist regarding the effects of transitioning between different enteral formulations and their impact on nutritional markers over time. Therefore, the present study aimed to evaluate the association between HPFs and HDFs on anthropometric and biochemical parameters, including body weight, serum albumin and total protein. By comparing chronically ventilated patients receiving enteral nutrition maintained on HPFs, those transitioning to HPFs, those transitioning away from HPFs and those transitioned to HDFs, this study sought to better characterize the role of formula composition and caloric density in clinical nutritional management.

## 2. Materials and Methods

### 2.1. Study Design and Population

This retrospective study included adult patients who were chronically ventilated and receiving total long-term enteral nutrition. This study was designed as a retrospective observational study conducted at a single center (Bet Hadar Ashdod Nursing & Rehabilitation Medical Center, Ashdod, Israel), reflecting real-world clinical practice, between July 2023 and April 2025. Adult patients (≥18 years) who received enteral nutrition during hospitalization were eligible for inclusion. Underlying conditions included chronic respiratory failure, neurological disorders and post-critical illness states. This study included patients receiving an HPF (Easy Daily) (Easy Line, Caesarea, Israel) or HDF (Easy Meal-k 2) (Easy Line, Caesarea, Israel). Patients were included if they had documented use of enteral nutrition for at least 6 months and available anthropometric and biochemical data at baseline and follow-up. Exclusion criteria included missing data, short hospitalization period, parenteral nutrition only, incomplete laboratory data, insufficient duration of enteral feeding for evaluation (at least 6 months), or significant clinical events (e.g., acute infection) likely to affect nutritional biomarkers during the study period. Patients were categorized into four groups based on their enteral nutrition regimen during the 12 months preceding data collection, with specific emphasis on the most recent 6-month period: (1) Continuous HPF group: Patients received an HPF continuously for the entire 6-month period prior to data collection. (2) Transition to HPF group: Patients received standard enteral nutrition for the initial 6 months, followed by a transition to an HPF for the subsequent 6 months leading up to data collection. (3) Transition from HPF group: Patients received an HPF for the initial 6 months, followed by a transition to an alternative enteral formula for the subsequent 6 months prior to data collection. (4) Transition to HDF group: Patients received standard enteral nutrition for the initial 6 months, followed by a transition to an HDF for the subsequent 6 months leading up to data collection. All feeding strategies were implemented based on clinical judgment, taking into account patients’ nutritional requirements, tolerance and overall clinical condition.

### 2.2. Nutritional Intervention

The HPF (Easy Daily, Easy line, Caesarea, Israel) is a high-protein formula for enteral nutrition intended to provide balanced caloric intake with an emphasis on protein adequacy. The HDF (Easy Meal-k 2, Easy Line, Caesarea, Israel) is a high-caloric-density enteral formula designed to provide increased energy delivery in lower volumes, which may be advantageous in patients with fluid restrictions or reduced feeding tolerance. The HPF was characterized by a higher protein content (~6.5 g/100 mL) and standard caloric density (~1.3 kcal/mL). The HDF provided a higher caloric density (~2 kcal/mL) with a moderate protein content (8 gr/100 mL). Enteral nutrition was initiated according to institutional protocols. Administration protocols were determined by the clinical team based on individual patient needs. The formulas were administered according to clinical practice, including continuous or intermittent feeding via enteral access. Caloric goals and infusion rates were determined by the treating clinical team based on estimated energy requirements, consistent with established clinical nutrition guidelines [14]. Nutritional intake was not standardized and was adjusted individually based on clinical judgment.

### 2.3. Data Collection

Demographic and clinical data were extracted from medical records. Anthropometric and biochemical parameters were assessed at two time points: baseline (prior to initiation or transition of feeding strategy) and follow-up (approximately 6 months of nutritional intervention). The primary anthropometric parameter measured was body weight (kg). Biochemical parameters included serum albumin (g/dL) and total protein (g/dL), which were used as indicators of nutritional and protein status. The primary outcomes were changes in body weight, serum albumin and total protein levels between baseline and follow-up within each group. Secondary analyses included comparison of trends across the four feeding strategies to evaluate differential effects of the HPF and HDF depending on their mode of use.

### 2.4. Statistical Analysis

Continuous variables were expressed as mean ± standard deviation. Paired comparisons between baseline and follow-up values within each group were performed using the appropriate statistical tests (paired *t*-test or non-parametric equivalent, depending on data distribution). Continuous variables were assessed for normality using the Shapiro–Wilk test. A *p*-value of <0.05 was considered statistically significant. All analyses were conducted to assess within-group changes rather than direct between-group comparisons given the observational nature of the study and potential baseline differences. Statistical analyses were performed using JMP software (version JMP Student Edition 18, SAS Institute, Cary, NC, USA). Given the observational design, the analyses were not intended to establish causal relationships. 

## 3. Results

### 3.1. Study Population

The study population consisted of chronically ventilated patients with heterogeneous underlying conditions, including chronic respiratory failure and neurological impairment. The patients receiving enteral nutrition were categorized into four groups: continuous HPF feeding (*n* = 13), transition to HPF (*n* = 20), transition from HPF (*n* = 9), and transition to HDF (*n* = 8) (Table 1). The mean age differed modestly across groups, with patients in the continuous HPF group being older (73.6 ± 19.1 years) compared to the transition to HPF (69.4 ± 16.8 years), transition from HPF (64.3 ± 13.4 years) and transition to HDF groups (62.0 ± 14.4 years). The sex distribution was relatively balanced across groups, although the HDF group included a higher proportion of males (Table 1). Baseline BMI values were broadly comparable among the HPF groups (28.0 ± 4.2, 26.0 ± 5.0, and 27.4 ± 5.7 kg/m^2^, respectively), while patients in the HDF group had a lower mean BMI (20.8 ± 1.5 kg/m^2^), suggesting a more nutritionally compromised population at baseline. Similarly, baseline body weight was lower in the HDF group (61.4 ± 9.5 kg) compared to the HPF groups (Table 1). Serum albumin levels were generally comparable across groups, although slightly lower variability was observed in the transition-from-HPF group (3.4 ± 0.2 g/dL). Total protein levels were similar across all groups (Table 1). Given the observational design and some baseline differences, analyses focused on within-group changes rather than direct between-group comparisons.

### 3.2. Effect of Continuous HPF Administration on Body Weight, Albumin and Total Protein

**Figure 1:**
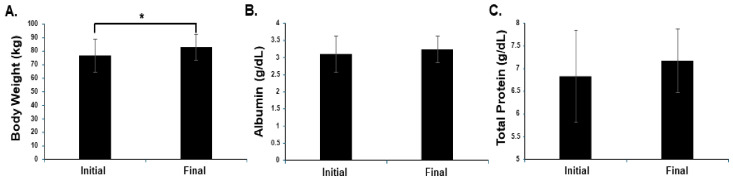
Effect of continuous HPF feeding on anthropometric and biochemical parameters. (**A**) Body weight. (**B**) Serum albumin. (**C**) Total protein. Changes between baseline and follow-up are shown. Data are presented as mean values. Asterisks indicate statistically significant differences (* *p* < 0.05).

Following continuous HPF administration, changes were observed in anthropometric and biochemical parameters. Body weight increased significantly from 76.53 kg at baseline to 82.81 kg (*p* = 0.0229) (Figure 1A), indicating an overall improvement in caloric status. Serum albumin increased modestly from 3.11 g/dL to 3.24 g/dL. However, this change did not reach statistical significance (*p* = 0.2802) (Figure 1B). Total protein increased from 6.83 g/dL to 7.18 g/dL but did not reach statistical significance (*p* = 0.1121) (Figure 1C). Overall, continuous HPF administration was associated with a significant increase in body weight and modest, non-significant improvements in protein-related biochemical markers.

### 3.3. Effect of Transition to HPF on Body Weight, Albumin and Total Protein

**Figure 2:**
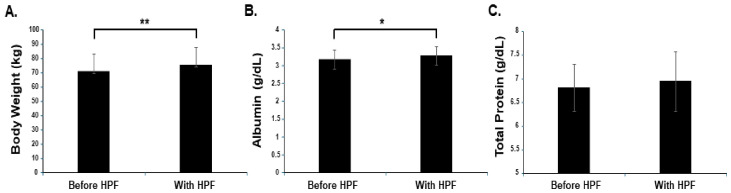
Effect of transition to HPF on anthropometric and biochemical parameters. (**A**) Body weight. (**B**) Serum albumin. (**C**) Total protein. Changes between baseline and follow-up are shown. Data are presented as mean values. Asterisks indicate statistically significant differences (* *p* < 0.05, ** *p* < 0.01).

Following transition to an HPF, changes were observed in anthropometric and biochemical parameters. Body weight increased significantly from 70.82 kg at baseline to 75.35 kg (*p* = 0.00019) (Figure 2A), indicating an overall improvement in caloric status. Serum albumin increased from 3.18 g/dL to 3.28 g/dL, reaching statistical significance (*p* = 0.0280) (Figure 2B). Total protein increased from 6.80 g/dL to 6.94 g/dL but did not reach statistical significance (*p* = 0.0968) (Figure 2C). Overall, transition to an HPF was associated with a significant increase in body weight and serum albumin, along with a modest, non-significant increase in total protein.

### 3.4. Effect of Transition from HPF on Body Weight, Albumin and Total Protein

**Figure 3:**
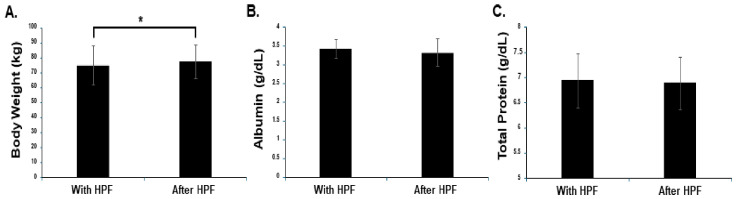
Effect of transition from HPF on anthropometric and biochemical parameters. (**A**) Body weight. (**B**) Serum albumin. (**C**) Total protein. Changes between baseline and follow-up are shown. Data are presented as mean values. Asterisks indicate statistically significant differences (* *p* < 0.05).

Following transition from an HPF to alternative formulas, changes were observed in anthropometric and biochemical parameters. Body weight increased significantly from 74.65 kg at baseline to 77.31 kg (*p* = 0.0479) (Figure 3A), indicating an overall improvement in caloric status. In contrast, serum albumin decreased slightly from 3.42 g/dL to 3.32 g/dL, although this change did not reach statistical significance (*p* = 0.2138) (Figure 3B). Total protein remained essentially unchanged, with values of 6.94 g/dL at baseline and 6.89 g/dL post-intervention (*p* = 0.5859) (Figure 3C). Overall, transition from an HPF was associated with a modest increase in body weight without corresponding improvements in protein-related biochemical markers.

### 3.5. Effect of Transition to HDF on Body Weight, Albumin and Total Protein

**Figure 4:**
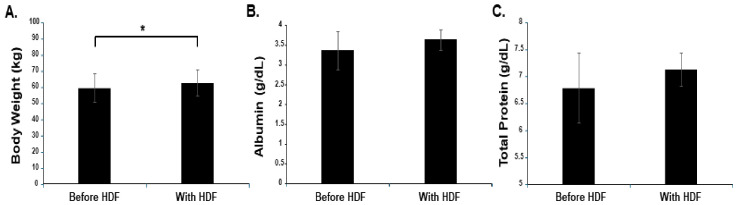
Effect of transition to HDF on anthropometric and biochemical parameters. (**A**) Body weight. (**B**) Serum albumin. (**C**) Total protein. Changes between baseline and follow-up are shown. Data are presented as mean values. Asterisks indicate statistically significant differences (* *p* < 0.05).

Following the introduction of an HDF, changes were observed in anthropometric and biochemical parameters. Body weight increased significantly from 59.51 kg at baseline to 62.57 kg after HDF administration (*p* = 0.0389) (Figure 4A), indicating an overall improvement in caloric status. Serum albumin increased from 3.36 g/dL to 3.63 g/dL. However, this change did not reach statistical significance (*p* = 0.0869) (Figure 4B). Total protein increased from 6.78 g/dL to 7.12 g/dL and approached statistical significance (*p* = 0.05) (Figure 4C), suggesting a trend toward improved protein status following HDF administration. Overall, HDF administration was associated with a significant increase in body weight and favorable trends in protein-related biochemical markers.

## 4. Discussion

This study evaluated the relationship between high-protein and high-density enteral nutrition strategies and changes in anthropometric and biochemical parameters in chronically ventilated patients. Our findings demonstrate that while body weight increased across all groups, improvements in biochemical markers, particularly serum albumin, were most pronounced in patients who transitioned to an HPF. These results highlight the importance of both the composition of enteral formulas and the clinical context in which they are introduced.

The consistent increase in body weight across all groups suggests that overall caloric provision was adequate regardless of the specific formula used. This observation is supported by clinical guidelines and primary studies demonstrating that appropriately administered enteral nutrition can meet energy requirements and promote weight maintenance or gain in critically ill and mechanically ventilated patients [1,4,5]. Prospective and observational studies have shown that achieving prescribed caloric targets is associated with improved energy balance and attenuation of weight loss [15]. In particular, adequate enteral nutrition has been associated with improved weight stability and clinical outcomes compared to insufficient intake [15,16,17]. Our findings are consistent with these reports, as patients with higher energy intake demonstrated greater weight stability. However, in contrast to controlled trials with more homogeneous populations, our cohort included patients with diverse chronic conditions, which may explain the variability observed in outcomes. These differences highlight the importance of individualized nutritional strategies in complex, chronically ventilated populations. Notably, patients receiving HDFs also demonstrated significant weight gain, supporting the role of high-density formulations in improving caloric delivery [10], particularly in patients with feeding limitations or lower baseline BMI. Nevertheless, changes in body weight and BMI must be interpreted cautiously, as they may be influenced by fluid balance, edema, and other non-nutritional factors.

Despite uniform improvement in body weight, changes in biochemical markers were more variable. Only patients who transitioned to an HPF demonstrated a statistically significant increase in serum albumin, suggesting that increased protein provision may be particularly beneficial when introduced as a targeted intervention. This finding is consistent with prior literature indicating that protein-enriched formulas can improve visceral protein markers in patients with baseline deficiencies [18,19]. However, albumin is a negative acute-phase reactant and is strongly influenced by inflammation, hydration status and underlying disease [11,12]. In chronically ventilated patients, changes in albumin are therefore more likely to reflect variations in inflammatory status and clinical stability rather than the isolated effect of protein intake.

Patients maintained on continuous HPF feeding experienced significant weight gain without corresponding improvements in biochemical markers, which may reflect a state of nutritional stability in which the baseline protein intake was already sufficient. Similarly, patients transitioned away from an HPF demonstrated continued weight gain but a slight decline in albumin, suggesting a potential dissociation between caloric adequacy and protein status. This observation is supported by evidence indicating that enteral formulas differ not only in protein quantity but also in protein quality and bioavailability, which can influence nitrogen balance and protein metabolism independently of caloric intake [20]. Clinical and metabolic studies have shown that protein-enriched formulas, particularly those enriched with essential amino acids or whey protein, are associated with improved nitrogen retention and enhanced protein synthesis [21,22]. These effects may be especially relevant in critically ill patients with increased catabolic demand and altered protein metabolism [7]. In this context, protein composition may play a critical role in determining metabolic response and recovery of protein-related biomarkers.

The findings from the HDF group provide additional insight into the role of caloric density in nutritional management. While the HDF was effective in promoting weight gain, its impact on protein-related biochemical markers was more modest. This may reflect differences in macronutrient composition, lower absolute protein intake, or underlying clinical characteristics of this group, including lower baseline BMI and protein intake. These results suggest that while high-density formulas are effective for improving energy balance, they may need to be complemented with adequate protein provision to optimize overall nutritional status [5,20].

Across all groups, total protein levels showed limited sensitivity to change despite trends toward improvement in some subgroups. This is consistent with evidence indicating that total serum protein is influenced by multiple physiological factors and may not reliably reflect short-term nutritional interventions [23]. In critically ill and chronically ventilated patients, total protein levels are affected by hydration status, inflammation, hepatic protein synthesis and protein redistribution between compartments [23,24]. Acute-phase responses and fluid shifts can significantly alter circulating protein concentrations independent of nutritional intake [24]. Furthermore, the relatively long half-life of circulating proteins and the prioritization of acute-phase protein synthesis during illness limit responsiveness to short-term nutritional changes [14]. The lack of a clear association between protein intake and total protein levels in our cohort likely reflects these physiological influences rather than inadequate nutritional delivery. This dissociation between anthropometric and biochemical outcomes underscores the need for a comprehensive approach to nutritional assessment.

A key strength of this study is its evaluation of real-world enteral nutrition practices, including transitions between different formula types. This provides novel insight into how adjustments in nutritional strategies may influence patient outcomes over time. However, several limitations should be acknowledged. First, its retrospective design limits the ability to infer causality and introduces potential selection bias. Second, the relatively small sample size, along with unequal group sizes, may reduce the statistical power and generalizability of the findings. In addition, baseline differences between groups, particularly in age and body mass index (BMI), may have influenced the observed outcomes. The study population was also heterogeneous with respect to underlying chronic conditions, which may confound the interpretation of results. Importantly, no adjustment was made for disease severity, a factor that could significantly impact clinical and nutritional outcomes. The availability of inflammatory markers was limited, restricting a more comprehensive assessment of the patients’ inflammatory status. Finally, the use of albumin and BMI as surrogate nutritional markers has inherent limitations, as both are influenced by fluid balance and chronic illness, potentially reducing their accuracy in reflecting true nutritional status [23].

Despite these limitations, this study provides evidence that transitioning to HPFs may confer measurable benefits in nutritional status, particularly in terms of protein-related parameters. These findings are consistent with the broader literature supporting the role of optimized enteral formulations in improving clinical outcomes among nutritionally at-risk patients [17,25]. Further research is needed to confirm these observations and to better define the patient populations most likely to benefit from such interventions.

## 5. Conclusions

In this retrospective study, enteral nutrition strategies were associated with changes in anthropometric and biochemical parameters in chronically ventilated patients. Both HPFs and HDFs were associated with significant improvements in body weight, highlighting their effectiveness in supporting caloric adequacy. However, improvements in protein-related biochemical markers were most evident following transition to an HPF, while HDF administration demonstrated favorable but non-significant trends. These findings emphasize that caloric density and protein content play distinct but complementary roles in enteral nutrition, and that optimal nutritional strategies should be tailored to individual patient needs. In addition, these changes are likely influenced by underlying clinical condition, chronic inflammation, and fluid status rather than nutrition alone. Future prospective studies with standardized feeding protocols, larger sample sizes, and longer follow-ups are needed to better define optimal protein and energy targets and to evaluate individualized nutrition strategies across different patient populations. Additionally, the inclusion of objective functional markers such as handgrip strength and mid-upper arm circumference, along with biochemical indicators like prealbumin and nitrogen balance, would provide a more comprehensive assessment of nutritional outcomes.

## Figures and Tables

**Table 1:** Baseline Characteristics of the Study Population.

	Continuous HPF (*n* = 13)	Transition to HPF (*n* = 20)	Transition from HPF (*n* = 9)	Transition to HDF (*n* = 8)
Age (years)	73.6 ± 19.1	69.4 ± 16.8	64.3 ± 13.4	62.0 ± 14.4
Sex (male/female)	6/7	11/9	4/5	7/1
BMI (kg/m^2^)	28.0 ± 4.2	26.0 ± 5.0	27.4 ± 5.7	20.8 ± 1.5
Body weight (kg)	76.5 ± 12.2	69.7 ± 12.9	73.2 ± 11.9	61.4 ± 9.5
Serum albumin (g/dL)	3.1 ± 0.5	3.1 ± 0.4	3.4 ± 0.2	3.1 ± 0.8
Total protein (g/dL)	6.8 ± 1.0	6.6 ± 0.5	7.0 ± 0.4	6.6 ± 0.9
Daily protein intake (g) *	14.6 ± 13.5	17.9 ± 11.1	28.0 ± 6.1	5.2 ± 7.8

Values are presented as mean ± standard deviation. BMI—body mass index. HPF—high-protein formula. HDF—high-density formula. * Daily protein intake refers to the amount of protein provided in addition to that contained in the enteral formula.

## Data Availability

The original contributions presented in this study are included in the article. Further inquiries can be directed to the corresponding author.
